# Whole Genome Sequencing of “Mutation-Negative” Individuals With Cornelia de Lange Syndrome

**DOI:** 10.1155/humu/4711663

**Published:** 2025-01-30

**Authors:** Morad Ansari, Mihail Halachev, David Parry, Jose L. Campos, Elston N. D'Souza, Christopher Barnett, Andrew O. M. Wilkie, Angela Barnicoat, Chirag V. Patel, Elena Sukarova-Angelovska, Katta M. Girisha, Helen V. Firth, Katrina Prescott, Louise C. Wilson, Meriel McEntagart, Rosemarie Davidson, Sally Ann Lynch, Shelagh Joss, Simon T. Holden, Wayne K. Lam, Sanjay M. Sisodiya, Andrew J. Green, Gemma Poke, Nicola Whiffin, David R. FitzPatrick, Alison Meynert

**Affiliations:** ^1^South East Scotland Genetic Service, Western General Hospital, Edinburgh, UK; ^2^MRC Human Genetics Unit, University of Edinburgh, Edinburgh, UK; ^3^Wellcome Centre for Human Genetics, University of Oxford, Oxford, UK; ^4^Faculty of Health and Medical Sciences, University of Adelaide, Adelaide, South Australia, Australia; ^5^MRC Weatherall Institute of Molecular Medicine, University of Oxford, John Radcliffe Hospital, Oxford, UK; ^6^Craniofacial Unit, Oxford University Hospitals NHS Foundation Trust, John Radcliffe Hospital, Oxford, UK; ^7^Clinical Genetics, Great Ormond Street Hospital for Children NHS Foundation Trust, London, UK; ^8^Genetic Health Queensland, Royal Brisbane & Women's Hospital, Brisbane, Queensland, Australia; ^9^Clinical Genetics, University Pediatric Clinic, Ss. Cyril and Methodius University in Skopje, Skopje, North Macedonia; ^10^Department of Medical Genetics, Kasturba Medical College, Manipal, Manipal Academy of Higher Education, Manipal, India; ^11^Clinical Genetics, Addenbrooke's Hospital, Cambridge University Hospitals, Cambridge, UK; ^12^Yorkshire Regional Genetics Service, Chapel Allerton Hospital, Leeds, UK; ^13^Medical Genetics, St. George's University Hospitals NHS FT, London, UK; ^14^West of Scotland Regional Genetics Service, Queen Elizabeth University Hospital, Glasgow, UK; ^15^Department of Clinical Genetics, Children's Health Ireland, University College Dublin School of Medicine, Dublin, Ireland; ^16^Department of Clinical and Experimental Epilepsy, UCL Queen Square Institute of Neurology, London, UK; ^17^Chalfont Centre for Epilepsy, Chalfont Saint Peter, UK; ^18^Central Hub, Genetic Health Service, Wellington, New Zealand

## Abstract

This study was aimed at assessing the diagnostic utility of whole genome sequence analysis in a well-characterised research cohort of individuals referred with a clinical suspicion of Cornelia de Lange syndrome (CdLS) in whom prior genetic testing had not identified a causative variant. Short-read whole genome sequencing was performed on 195 individuals from 105 families, 108 of whom were affected. 100/108 of the affected individuals had prior relevant genetic testing, with no pathogenic variant being identified. The study group comprised 42 trios in which both parental samples were available for testing (42 affected individuals and 126 unaffected parents), 61 singletons (unrelated affected individuals), and two families with more than one affected individual. The results showed that 32 unrelated probands from 105 families (30.5%) had likely causative coding region-disrupting variants. Four loci were identified in > 1 proband: *NIPBL* (10), *ANKRD11* (6), *EP300* (3), and *EHMT1* (2). Single variants were detected in the remaining genes (*EBF3*, *KMT2A*, *MED13L*, *NLGN3*, *NR2F1*, *PHIP*, *PUF60*, *SET*, *SETD5*, *SMC1A*, and *TBL1XR1*). Possibly causative variants in noncoding regions of *NIPBL* were identified in four individuals. Single de novo variants were identified in five genes not previously reported to be associated with any developmental disorder: *ARID3A*, *PIK3C3*, *MCM7*, *MIS18BP1*, and *WDR18*. The clustering of de novo noncoding variants implicates a single upstream open reading frame (uORF) and a small region in Intron 21 in *NIPBL* regulation. Causative variants in genes encoding chromatin-associated proteins, with no defined influence on cohesin function, appear to result in CdLS-like clinical features. This study demonstrates the clinical utility of whole genome sequencing as a diagnostic test in individuals presenting with CdLS or CdLS-like phenotypes.

## 1. Introduction

Cornelia de Lange syndrome (CdLS) is a severe multisystem disorder characterised by malformations of the limb and diaphragm, prenatal-onset growth failure, gastrointestinal dysfunction, neurodevelopmental problems, and characteristic facies [1]. Most typical CdLS is caused by heterozygous loss-of-function (LOF) mutations in the gene encoding the cohesin loader, NIPBL [2, 3]. Almost all *NIPBL* mutations causing typical CdLS occur de novo, with ~30% being postzygotic mosaics [4, 5]. Over the last 20 years, mutations in genes encoding components of the cohesin ring (*SMC1A* [6], *SMC3* [7], and *RAD21* [8]) or proteins required for normal DNA-cohesin interaction (*HDAC8* [9]) have been identified in individuals with atypical forms of CdLS. More recently, individuals with a provisional diagnosis of CdLS have been reported with de novo mutations in genes encoding chromatin-associated proteins with no direct role in cohesin function, for example, *ANKRD11*, *SETD5*, and *KMT2A* [10].

Here, we present an analysis of short-read whole genome sequencing (WGS) on blood- or saliva-derived DNA to analyse a cohort of 108 affected individuals from 105 families with a provisional clinical diagnosis of CdLS or a CdLS-like disorder. Almost all of these individuals had previously been screened negative for mutations in known CdLS genes [5]. The results provide further support for *NIPBL* as the dominant locus in CdLS. We have identified clustered de novo mutations affecting the noncoding regions of *NIPBL* and balanced and unbalanced intragenic structural variants (SVs). Causative variants disrupting the coding region were identified in 14 other genes, almost all encoding chromatin-associated proteins. We also identified single de novo variants in five genes without strong prior evidence of association with developmental disorders (DDs).

## 2. Materials and Methods

### 2.1. Research Participant Information

The data presented in this study are derived from DNA samples and clinical information from research participants who have consented to be involved in the CdLS study managed by the MRC Human Genetics Unit in collaboration with the CdLS Foundation of the United Kingdom and Ireland (http://www.cdls.org.uk). The cohort consists of 299 affected individuals with 293 unaffected relatives. These samples are held with the consent of the families obtained using a process approved by the United Kingdom multicentre research ethics committee (MREC) for Scotland (Committee A) for WGS (04:MRE00/19; the genetics of brain growth and development). All affected individuals have been examined by an experienced clinical geneticist. Potentially diagnostic results from the research sequencing are communicated to the referring clinicians for validation in the local genetic diagnostic laboratories.

### 2.2. DNA Sequencing, Alignment, and Variant Calling

WGS of the quality-checked DNA was performed at Edinburgh Genomics, University of Edinburgh. FASTQ alignment used BCBio-Nextgen (0.9.7) for bam file preparation; bwa mem (v0.7.13) aligned reads to GRCh38 reference genome employing alt, decoy, and HLA sequences. Duplicated fragments were marked using samblaster (v0.1.22) and indel realignment, and base recalibration was performed using GATK 3.4 to create a final gVCF file.

### 2.3. Diagnostic Variant Filtering

We used a genome-wide approach to identify de novo mutations in the trio samples using both cyvcf2 [11] and VASE (https://github.com/david-a-parry/vase). All probands were also screened for plausibly causative variants in known DD genes using the G2P-VEP plugin with Ensembl VEP [12]. From all the variants identified in an individual, we selected only those that are rare, predicted to be functional, and potentially relevant to DDs by using the G2P plugin (10.1038/s41467-019-10016-3) in VEP (release 90.1, doi:10.1186/s13059-016-0974-4) and the DD gene panel (https://www.ebi.ac.uk/gene2phenotype/downloads, accessed 11/06/2018). In short, we extracted only variants satisfying the inheritance requirements of the genes in the DD gene panel, with minor allele frequency (MAF) in public databases < 0.0001 for monoallelic and X-linked genes and MAF < 0.005 for biallelic genes. We filtered to include only variants annotated by VEP to have one of the following consequences: stop gained, stop lost, start lost, frameshift variant, inframe insertion/deletion, missense variant, coding sequence variant, initiator codon variant, transcript ablation, transcript amplification, protein-altering variant, splice donor/acceptor variant (i.e., canonical splice site), or splice region variant (i.e., either within 1–3 bases of the exon or 3–8 bases of the intron). Integrative Genomics Viewer (IGV) plots of each candidate variant were generated from the trio, singleton, and multiplex families.

### 2.4. SV Analysis

De novo SVs were called from the bam files in each trio using a paired-end and split read method (Manta; https://github.com/Illumina/manta) and a coverage-based method (Canvas; https://github.com/Illumina/canvas). Each entry in the candidate SVs was associated with an image visualising the coverage and alignment within the trio.

### 2.5. Annotating Variants in Untranslated Regions

De novo variants identified in the 5⁣′untranlsated region (5⁣′UTR) of *NIPBL* were annotated with UTRannotator (https://academic.oup.com/bioinformatics/article/37/8/1171/5905476). We also annotated all variants in ClinVar (downloaded on 30/04/2022) and gnomAD v3.1.1 within the 5⁣′UTR as defined by the MANE Select [13] transcript (chr5:36876769-36877178 and chr5:36953618-36953696 on GRCh38). We retained all variants with an annotation indicative of creating an upstream start-codon (uAUG-gained) or disrupting a predicted upstream open reading frame (uORF; uAUG-lost, uSTOP-lost, uSTOP-gained, or uFrameshift). ClinVar variants were further filtered to those classified as pathogenic, likely_pathogenic, or pathogenic/likely_pathogenic. Finally, we searched the literature for any additional 5⁣′UTR variants identified in individuals with CdLS. The strength of the Kozak consensus surrounding each uAUG was defined as either weak, moderate, or strong, as has been done previously.

### 2.6. Generation of Protein Images

The R package drawProteins [14] was used to generate cartoons of the domain structure of proteins encoded by the MANE Select [13] transcript using data obtained from UniProt [15]. The position of the variants predicted to affect the coding region was added using simple R commands using the R package ggplot2 [16].

## 3. Results

### 3.1. Case Selection

This study was designed to assess short-read WGS as a diagnostic tool in CdLS. Following a review of DNA quality and prior molecular genetic analysis in the 299 affected individuals participating in the MRC HGU CdLS cohort, we identified 100 affected individuals who had screened negative for mutations in the core CdLS genes (*NIPBL*, *SMC1A*, *SMC3*, *RAD21*, and *HDAC8*) [5] and eight probands who had no prior screening of the CdLS genes. Growth details and clinical synopsis relating to the affected individuals discussed below were available for a subset of cases and are provided in Supporting Information 1. The WGS cohort consisted of 61 singletons, 42 trios, and two quads (one affected sib pair with both unaffected parents and one affected sib pair with an affected and an unaffected parent), for a total of 195 individuals, 108 of whom were affected.

### 3.2. Variant Filtering

WGS reads were generated on 195 individuals and processed using the MRC Human Genetics Unit pipeline (see the Materials and Methods section). Since our primary aim is molecular diagnoses of affected individuals, the analyses focussed on identifying moderate or high-impact rare variants that occurred (1) using trio and quad families to identify de novo variants using a combination of cyvcf2 and VASE and (2) screening known DD genes in all individuals using the G2P-VEP plugin with the DDG2P dataset. We identified 60 candidates of monoallelic (heterozygous or hemizygous) variants in 54 probands that survived our filtering (see the Materials and Methods section; Supporting Information 2). No plausible biallelic genotypes survived filtering. Thirty-two variants in 32 probands were scored as pathogenic or likely pathogenic (P/LP) using ACMG criteria [17, 18]. Six variants in four probands were identified in the noncoding regions of *NIPBL*. Five de novo variants in five probands were identified in genes not previously associated with DDs.

### 3.3. P/LP Variants

**Figure 1:**
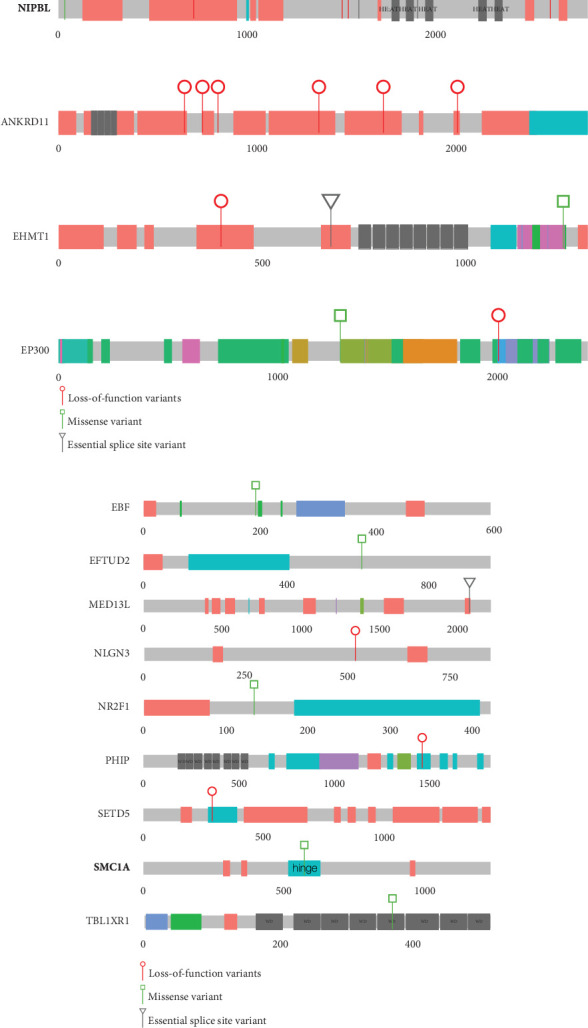
Pathogenic or likely pathogenic variants in known developmental disorder loci. This figure shows cartoons of 13 different proteins encoded by the loci in which causative heterozygous variants have been identified in this study. Each of these loci are known cause of developmental disorders. The proteins in bold script have a direct role in mediating the normal function of cohesin. (a) Four proteins in which variants in > 1 unrelated affected individual have been identified. The position and type of the variants are indicated using the key below this panel. (b) Proteins, mutation type, and position of the variants that have been identified in a single proband. The domain name is indicated when a missense variant lies within the domain.

**Figure 2:**
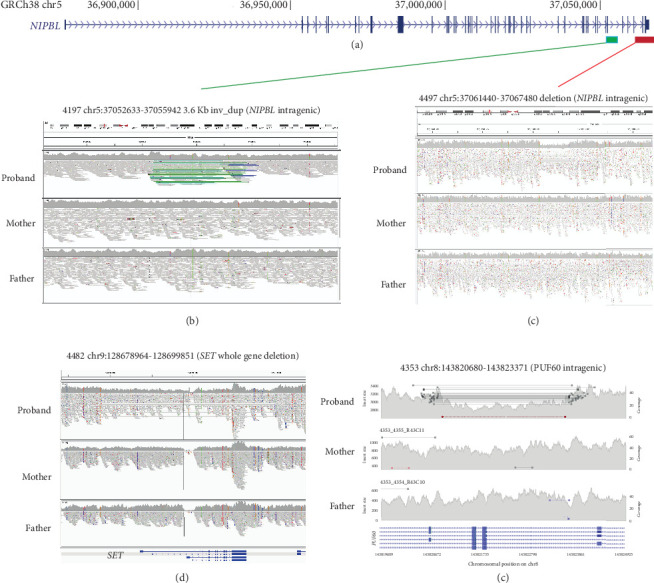
Causative structural variants. (a) Cartoon of the genomic structure of *NIPBL* coloured bars indicating the position of the structural variants shown in (b) and (c). (b) IGV plot of Proband 4197 and their parents showing a region of Chromosome 5. The green lines on the proband IGV plot indicate an inverted segment of chromosome with the blue lines representing a possible duplicated region (the coverage graph does not support this increased copy number). The inversion is predicted to encompass *NIPBL* Exons 42 and 43 and disrupt the open reading frame. (c) IGV plot of Proband 4497 and their parents. A heterozygous de novo deleted region is indicated by the drop in coverage in the proband and the grey lines on the IGV plot indicating paired end reads that cover the deletion breakpoints. This deletion encompasses Exons 46 and 47 which encode the most C-terminal region of NIPBL. (d) The IGV plot of Proband 4482 and their parents indicate a de novo deletion encompassing the whole *SET* gene. (e) The read plot of Proband 4353 and their parents indicating a de novo intragenic deletion involving the *PUF60* gene (plotted using samplot [https://github.com/ryanlayer/samplot]).

Heterozygous LOF mutations in the coding regions of *NIPBL* are, by far, the most common class of causative variants associated with CdLS [2, 3, 5, 19]. We identified 10 P/LP monoallelic *NIPBL* variants in 10 different probands, including two mosaic cases (Table 1 and Figure 1(a); Supporting Information 5). Eight of these could be shown to have occurred de novo (Table 1), and for two probands, the parental samples were not available for testing. 9/10 represent clear LOF variants: one stop gain (4445), three frameshifts (3616, 5263, and 5651), three essential splice sites (4536, 4691, and 5320), one disruptive intragenic inversion (4197, Figure 2(b)), and one intragenic deletion removing the most 3⁣′ coding exons (4497, Figure 2(c)). We also identified a de novo missense variant (p.(Ala34Val)) in Proband 4281 within a region that mediates the interaction of NIPBL with MAU2 [20]. In silico predictors (SIFT: deleterious (0.01); PolyPhen: probably damaging (0.98); CADD 26.2; REVEL 0.59; SpliceAI ≤0.2) are broadly supportive of a deleterious effect.

Heterozygous LOF variants in *ANKRD11* are, most commonly, associated with KBG syndrome [21, 22], but a phenotypic overlap with CdLS has been recurrently reported [5, 23–25]. We identified six P/LP LOF variants in *ANKRD11* in six unrelated probands (Table 1 and Figure 1(a); Supporting Information 5). In three families, these variants arose de novo (4252, 4294, and 4753), but for the remaining probands (3379, 3471, and 4348), parental samples were not available. Plausibly causative heterozygous variants in *EP300* and *EHMT1* were identified in three (3037, 3188, and 3961) and two (4187 and 4462 [de novo]) probands, respectively (Figure 1(a) and Table 1). Variants in both loci have been previously reported in individuals with a clinical suspicion of CdLS [23, 26].

Single probands with P/LP variants in 11 additional genes (*EBF3*, *KMT2A*, *MED13L*, *NLGN3*, *NR2F1*, *PHIP*, *PUF60*, *SET*, *SETD5*, *SMC1A*, and *TBL1XR1*) are documented in Figure 1(b) and Table 2. The de novo heterozygous missense variant in the hinge domain of *SMC1A* identified in proband 5661 is typical of CdLS-associated variants in this gene [6, 27–29]. *KMT2A*, *MED13L*, *PHIP*, and *SETD5* would not commonly be referred to as CdLS genes, but the heterozygous LOF mutations identified in probands 3236, 3057, 4248, and 3036, respectively, are comparable to those previously reported in CdLS [10, 23]. The remaining six probands (4021, 4482, 4383, 3046, 4353, and 3035) have variants in genes that have not been implicated in CdLS before but are known to be associated with nonsyndromic (*NLGN3* [30] and *SET* [31]) and/or syndromic (*EBF3* [32], *NR2F1* [33], *PUF60* [34], and *TBL1XR1* [35, 36]) intellectual disability, respectively (proven de novo in 4482 and 4353). We could not determine whether these variants represent false positive, contributary, or fully explanatory molecular diagnoses for the CdLS-like phenotype in the probands.

No plausibly causative shared variants were detected in the families with > 1 affected individual.

### 3.4. Clustering of Noncoding Variants in *NIPBL*

**Figure 3:**
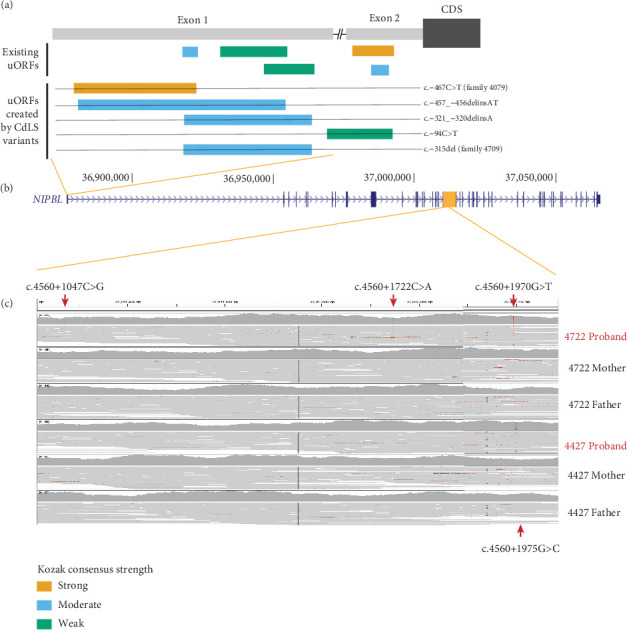
De novo variants affecting uORF structure and clustering in Intron 21 of *NIPBL*. (a) Cartoon of the position of the predicted uORFs in the 5⁣′UTR encoded by Exon 1 and Exon 2 of *NIPBL*, indicating the strength of the Kozak translational start sequence shown in yellow, blue, and green for strong, moderate, and weak, respectively. The positions of the de novo variants in Probands 4079 and 4709 and their predicted effects are also shown. (b) Cartoon of the NIPBL genomic structure derived from the UCSC Genome Browser indicating the position of the noncoding variants detailed in (a) and (c). (c) IGV snapshot of the ~1 kb interval containing the de novo, deep intronic variants identified in Intron 21. Three de novo variants (arrowed above the IGV plots) were identified in Proband 4722 and a single variant (arrowed below the IGV plot) in Proband 4427.

We identified two probands with de novo variants in the first exon of *NIPBL* (4079 and 4709; Table 3 and Figures 3(a) and 3(b)) which encode part of the 5⁣′UTR. The 5⁣′UTR of *NIPBL* contains five predicted uORFs, three within Exon 1 (Figure 3(a)). The de novo variant in proband 4079 (c.-467C>T) creates a novel upstream start codon (uAUG) into a strong Kozak consensus context, creating a new uORF that is 156 bps in length (Figure 3(a)). This variant was previously identified de novo in an individual with CdLS. Interestingly, two further variants reported in the literature [37] are predicted to also create uAUGs: (1) the c.-457_-456delinsAT variant identified de novo in a 15-year-old male with classic CdLS (moderate Kozak; 270 bp long uORF created), and (2) the c.-94C>T variant which creates a uAUG with a weak match to the Kozak consensus in a patient with a mild phenotype (Figure 3(a)). The de novo variant in proband 4709 (c.-315del) has not been observed previously. This variant deletes a single base of the 5⁣′UTR directly following the uAUG of an existing uORF with a moderate Kozak match. The variant shifts the reading frame of the uORF, extending it from 15 to 189 bps in length (Figure 3(a), Supporting Information 3). A different 5⁣′UTR variant reported previously (c.-321_-320delinsA) has the same predicted impact [38]. We searched the gnomAD v3.1.1 dataset for 5⁣′UTR variants with similar predicted effects (Supporting Information 4). Whilst two variants, each identified in a single gnomAD individual, create uAUGs, both have a weak match to the Kozak consensus. Six variants are predicted to shift the frame of an existing uORF, but the impacted uORFs also have a weak Kozak consensus, so they are unlikely to be strongly translated. The clustering and predicted consequence of 5⁣′UTR variants in CdLS patients suggests an important role for uORF regulation in NIPBL translation.

In Proband 4722, we identified three different de novo variants within a 1 kb region of Intron 21 (Table 3 and Figure 3(c)). In Proband 4427, we identified a single de novo variant (c.4560+1975G>C, Table 3 and Figure 3(c)) that is only five base pairs away from the most 3⁣′ 4722 variant (c.4560+1970G>T) within a short interspersed nuclear element (SINE) repeat element. None of these deep intronic variants are in gnomAD, and none show evidence of a deleterious effect on splicing, and each has a low CADD score (Table 3). We are currently unable to perform any functional analysis of this segment of Intron 21 and thus cannot predict a consequence for these variants.

### 3.5. De Novo Variants in Genes Not Previously Implicated in DDs

**Figure 4:**
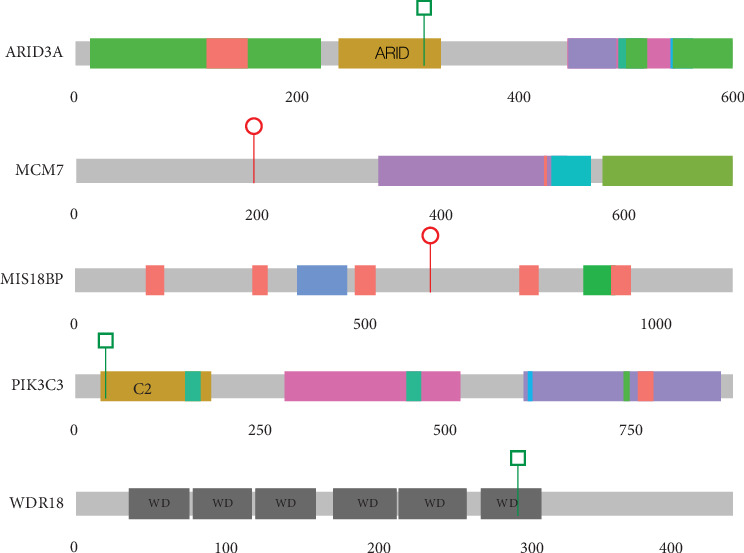
De novo protein-coding variants in genes not known to be associated with developmental disorders. This figure shows cartoons of five different proteins encoded by the loci in which de novo variants have been identified in this study. None of these loci are known causes of developmental disorders. The position and type of the variants are indicated using the key used in Figure 1. The domain name is indicated when a missense variant lies within the domain.

Following the IGV inspection of candidate de novo calls, five variants in five “novel” genes (i.e., not present in the DDG2P dataset) were identified in five different probands (Table 4 and Figure 4), including individual 4353, which also has a de novo intragenic deletion in *PUF60* (Table 2 and Figure 2), making it difficult to attribute any contribution of *MIS18BP1* to the phenotype. In Proband 4954, in silico predictions show only weak evidence of deleteriousness for the *WDR18* missense variant. Neither of these variants will be considered further. Of the remaining genes (*PIK3C3*, *MCM7*, and *ARID3A*), only *MCM7* (proband 4485) has any direct link to cohesin function. *MCM7* encodes a subunit of the replicative helicase MCM2-7 which is required for the loading of cohesin onto DNA during S-phase. *ARID3A* encodes a widely expressed transcription factor with roles in haematopoiesis, placental development, and mesoderm formation. *PIK3C3* encodes a component of the complex that catalyses phosphatidylinositol 3-phosphate formation. Mechanistically, this would not represent an obvious candidate gene for CdLS.

## 4. Discussion

Diagnostic genomic analysis of individuals with severe DDs can confidently identify genes with an important and nonredundant developmental role. It is reasonable to hypothesise that the identification of these products will indicate specific critical functions they mediate during embryogenesis and improve our understanding of developmental pathology. CdLS is very commonly described as a cohesinopathy [39] on the grounds of the phenotypic overlap of individuals with mutations in genes encoding the components of the cohesin ring and factors regulating its interaction with DNA. However, the very large number of different functions of cohesin somewhat limits our understanding of the specific disease mechanisms. It is not unreasonable to assume that identifying other disease loci with significant phenotypic overlap with CdLS may implicate perturbation of a subset of cohesin roles in the disease mechanism.

In every published CdLS cohort analysis, *NIPBL* is by far the most frequently mutated gene [3, 4, 23, 40–43]. We have previously reported a screen of a cohort of 168 individuals enriched with atypical CdLS [5]. 63/168 (37.5%) had coding region mutations in the known CdLS genes (*NIPBL*, *SMC1A*, *SMC3*, *RAD21*, and *HDAC8*), with 75% of the causal variants affecting the coding region of *NIPBL*. Given the almost universal association of severe typical CdLS with *NIPBL*, we estimated that ~20% of the unexplained cases are likely to be due to cryptic mutations or mosaicism at this locus. The current study was not designed to detect mosaicism, as it was based predominantly on the analysis of blood-derived DNA. However, one of the main advantages of diagnostic WGS is the identification of plausibly pathogenic variants in the noncoding regions of the transcription unit that would be missed in most whole exome sequencing (WES) analyses. In this regard, the two de novo variants identified in the 5⁣′UTR are particularly significant. Both have a plausible deleterious effect on translation [44], with predicted impacts similar to previously identified variants in the same region, suggesting that they are likely the causative variants in these individuals. Notably, these variants are > 300 bp upstream of the start of the *NIPBL* coding sequence and would not be captured using WES. Our analysis confirms an important role for uORF regulation of *NIPBL* in CdLS, suggesting that routine screening of the 5⁣′UTR is warranted in CdLS patients. The clustered de novo deep intronic variants that we identified in Intron 21 in two affected individuals are equally interesting but completely inexplicable from a mechanistic perspective. These have no predicted effect on splicing and alter bases that show no evolutionary conservation and, for the most clustered variant, lie within a SINE repeat (AluJb chr5:37012140-37012330, GRCh38). This region does show Transcription factor Occupancy prediction By Investigation of ATAC-seq Signal (TOBIAS) -corrected evidence of accessibility in inner cell mass cells derived from human embryos [45, 46], but we have no other direct evidence of *cis*-regulatory function. We do feel that these variants should be considered “of interest” but cannot yet be considered diagnostic.

In the same cohort analysis mentioned above [5], we also identified three individuals with heterozygous LOF mutations in *ANKRD11* who were, from a clinical perspective, no less typical than those with mutations in *HDAC8*, *RAD21*, and *SMC3*. Since then, many other loci have been reported as rarely causal in CdLS: *KMT2A* [23, 47], *SETD5* [23, 48], *EP300* [26], *MED13L* [23], *PHIP* [23], *AFF4* [49], *TAF6* [50], *MAU2* [51], *EHMT1* [23], and *BRD4* [52]. In our current study, we provide further support for the association of CdLS-like features with *ANKRD11*, *EP300*, *EHMT1*, *SETD5*, *MED13L*, and *PHIP* (Figures 1(a) and 1(b)). Additionally, we have identified P/LP variants in *EBF*, *EFTUD2*, *NLGN3*, *NR2F1*, *TBL1XR1* (Figure 1(b)), and *SET* (Figure 2) in known DD loci. Most of these genes encode chromatin-associated proteins, but except for MAU2 and BRD4, they provide no evidence of direct interaction with the cohesin system. Of the genes with de novo variants without known disease association, only *MCM7* encodes a protein with a direct link to cohesin. We have not yet found a satisfactory unifying explanation for the CdLS-like phenotypes that are associated with this set of genes. The general term transcriptomopathy [50] is useful conceptually but, like cohesinopathy, is too broad for detailed mechanistic use.

Given the expected and observed level of locus and allelic heterogeneity and phenotypic variability seen in individuals with a suspected diagnosis of CdLS, the present study was not designed to address specific genotype–phenotype associations. The collection, at scale, and appropriately consented to share of well-structured, comprehensive quantitative and categorical clinical data associated with the results of all molecular analyses will be required to discern such associations.

Further analysis of the mutation-negative cases with CdLS should, ideally, exclude postzygotic mosaic variants in *NIPBL* using analysis of DNA from a tissue such as uncultured skin. This would allow us to identify any false association in the existing data. There is a need for further experimental work focussed on identifying a functional link between NIPBL and the proteins encoded by the genes that have been recurrently identified in individuals with CdLS, most notably, ANKRD11.

## Figures and Tables

**Table 1:** Genes with pathogenic/likely pathogenic variants in > 1 proband.

**Family**	**Gene**	**DNM**	**GRCh38 locus and variant (MANE Select transcript)**	**Variant type**	**In gnomAD**
*NIPBL* (NM_133433.3)					
4281	*NIPBL*	Y	Chr5:36955508C>T c.101C>T p.(Ala34Val)	MIS	N
3616	*NIPBL*	?	Chr5:36985329_36985330del c.2149_2150del p.(Lys717GlufsT⁣^∗^2) mosaic: ref 32, alt 3	LOF	N
5651	*NIPBL*	Y	Chr5:37010177del c.4512del p.(Leu1504Phefs⁣^∗^85)	LOF	N
4445	*NIPBL*	Y	Chr5:37014728C>T c.4606C>T p.(Arg1536⁣^∗^) mosaic: ref 25, alt 3	LOF	N
4536	*NIPBL*	?	Chr5:37017018G>A c.4777-1G>A p.?	LOF (ESS)	N
4691	*NIPBL*	Y	Chr5:37022050G>C c.5329-1G>C p.?	LOF (ESS)	N
5320	*NIPBL*	Y	Chr5:37026228G>A c.5710-1G>A p.?	LOF (ESS)	N
5263	*NIPBL*	Y	Chr5:37060974del c.7816del p.(Ile2606Serfs⁣^∗^5)	LOF	N
4197	*NIPBL*	Y	chr5:37052633-37055017 and chr5:37055031-37055942~3.6 Kb SV inv or inv_dup in NIPBL ex42-43 region	LOF (SV)	N
4497	*NIPBL*	Y	chr5:37061440-37067480 deletion encompassing the last two coding exons (46 and 47) of NIPBL	LOF (SV)	N
*ANKRD11* (NM_013275.5)					
3379	*ANKRD11*	?	Chr16:89280521del c.6021delC p.(Phe2008Serfs⁣^∗^79)	LOF	N
3471	*ANKRD11*	?	Chr16:89284635_89284639del c.1903_1907del p.(Lys635Glnfs⁣^∗^26)	LOF	N
4252	*ANKRD11*	Y	Chr16:89281638_89281639insGC c.4903_4904insGC p.(Leu1635Argfs⁣^∗^52)	LOF	N
4294	*ANKRD11*	Y	Chr16:89282611G>A c.3931C>T p.(Arg1311⁣^∗^)	LOF	N
4348	*ANKRD11*	?	Chr16:89284130_89284134del c.2408_2412del p.(Lys803Argfs⁣^∗^5)	LOF	N
4753	*ANKRD11*	Y	Chr16:89284364_89284367del c.2175_2178del p.(Asn725Lysfs⁣^∗^23)	LOF	Y⁣^∗^
*EP300* (NM_001429.3)					
3037	*EP300*	?	Chr22:41177730_41177731del c.6019_6020del p.(Gln2007Valfs⁣^∗^65)	LOF	N
3188	*EP300*	?	Chr22:41162780G>C c.3728+1G>C p.?	LOF (ESS)	N
3961	*EP300*	?	chr22:41166649A>G c.3857A>G p.(Asn1286Ser) SpliceAI donor gain 0.92; *Δ*S donor loss 0.4	MIS	N
*EHMT1* (NM_024757.4)					
4187	*EHMT1*	?	Chr9:137752355dup c.1195dupC p.(Gln399Profs⁣^∗^14)	LOF	N
4462	*EHMT1*	Y	Chr9:137834502A>ATCGAGGCCGGCGA c.3695_3715dup p.(Leu1238_Gly1239insVEAGEQL)	MIS	N

*Note:* ?, inheritance status could not be determined; N, variant is not in gnomAD v2; Y⁣^∗^, a single allele is present in gnomAD, the IGV plot of the gnomAD variant call suggests this to be mosaic.

Abbreviations: DNM, de novo mutation; ESS, essential splice site variant; LOF, loss-of-function variant; MIS, missense variant; SV, structural variant; Y, yes.

**Table 2:** Genes with pathogenic/likely pathogenic variants in a single proband.

**Family**	**Gene**	**DNM**	**Variant (CRCh38)**	**Variant type**	**In gnomAD**
4383	*EBF3*	Y	Chr10:129877825C>A NM_001375380.1(EBF3):c.579G>T p.(Lys193Asn) REVEL0.437; SpliceAI≤0.2	MIS	N
**3236**	***KMT2A***	**?**	**Chr11:118484286dup NM_001197104.1(KMT2A):c.4190dup p.(Val1398Serfs∗9)**	**LOF**	**1ᵃ**
**3057**	***MED13L***	**?**	**Chr12:115966244C>A NM_015335.4(MED13L):c.6226-1G>T p.?**	**LOF (ESS)**	**N**
4021	*NLGN3*	?	ChrX:71167650G>A NM_181303.2(NLGN3):c.1553G>A p.(Trp518⁣^∗^) hemizygous	LOF	N
3046	*NR2F1*	?	Chr5:93585427G>A NM_005654.6(NR2F1):c.404G>A p.(Arg135His) REVEL0.962; SpliceAI≤0.2	MIS	N
**4248**	***PHIP***	**?**	**Chr6:78946244dup NM_017934.7(PHIP):c.4387dup p.(Arg1463Lysfs∗35)**	**LOF**	**N**
**4353**	***PUF60***	Y	**Chr8:143820938-143823597 deletes Exons 3 and 4 of PUF60**	**LOF (SV)**	**N**
4482	*SET*	Y	Chr9:128678964-128699851 deletion encompassing *SET*	LOF (SV)	N
**3036**	***SETD5***	**?**	**Chr3:9441638del NM_001080517.1(SETD5):c.856del p.(Leu286∗)**	**LOF**	**N**
**5661**	***SMC1A***	**Y**	**ChrX:53405788G>A NM_006306.4(SMC1A):c.1714C>T p.(Pro572Ser) REVEL0.86; SpliceAI≤0.2**	**MIS**	**N**
3053	*TBL1XR1*	?	Chr3:177038113G>T NM_024665.7(TBL1XR1):c.1107C>A p.(Asp369Glu) REVEL0.379; SpliceAI≤0.2	MIS	N

*Note:* The rows in bolded text are loci that have previously been reported to be implicated in the pathogenesis of CdLS and CdLS-overlapping phenotypes. ?, inheritance status could not be determined; N, variant is not in gnomAD v3.

Abbreviations: DNM, de novo mutation; ESS, essential splice site variant; LOF, loss-of-function variant; MIS, missense variant; SV, structural variant; Y, yes.

^a^A single heterozygous individual is present in gnomAD v3; however, read data is not available to assess. Variants are heterozygous unless stated otherwise.

**Table 3:** De novo noncoding variants in *NIPBL.*

**Family**	**Gene**	**DNM**	**GRCh38 coordinates**	**cDNA (NM_133433.4)**	**CADD**	**SpliceAI**	**Mutation type**	**In gnomAD**
4079	*NIPBL*	Y	Chr5:36876791C>T	c.-467C>T	20.1	≤ 0.2	NC (uORF)	N
4709	*NIPBL*	Y	Chr5:36876943del	c.-315del	19.7	≤ 0.2	NC (uORF)	N
4427	*NIPBL*	Y	Chr5:37012200G>C	c.4560+1975G>C	1.3	≤ 0.2	NC (int 21)	N
4722	*NIPBL*	Y	Chr5:37011272C>G	c.4560+1047C>G	0.6	≤ 0.2	NC (int 21)	N
		Y	Chr5:37011947C>A	c.4560+1722C>A	4.3	≤ 0.2	NC (int 21)	N
		Y	Chr5:37012195G>T	c.4560+1970G>T	1.5	≤ 0.2	NC (int 21)	N

*Note:* N, variant is not in gnomAD v3.

Abbreviations: DNMs, de novo mutations; int 21, Intron 21 of *NIPBL* gene; NC, noncoding variant; uORF, upstream open reading frame in 5⁣′UTR; Y, yes.

**Table 4:** De novo variants in genes not known to cause developmental disorders.

**Family**	**Gene**	**DNM**	**Variant(s) of note**	**Mutation type**	**In gnomAD**
3060	*PIK3C3*	Y	Chr18:41957625C>T NM_002647.4(PIK3C3):c.124C>T p.(Pro42Ser) REVEL 0.66; SpliceAI ≤0.2	MIS	N
4353^a^	*MIS18BP1*	Y	Chr14:45226740-45226750delinsAACC NM_018353.4(*MIS18BP1*):c.1833_1840+3delinsAACC p.(Lys612Thrfs⁣^∗^14)	LOF	N
4485	*MCM7* ^b^	Y	Chr7:100098712G>A NM_005916.5(MCM7):c.586C>T p.(Gln196⁣^∗^)	LOF	N
4847	*ARID3A*	Y	Chr19:964425G>A NM_005224.3(ARID3A):c.944G>A p.(Arg315Gln) REVEL 0.72; SpliceAI≤0.2	MIS	N
4954	*WDR18*	Y	Chr19:991291G>A NM_024100.4(WDR18):c.871G>A p.(Glu291Lys) REVEL 0.268; SpliceAI ≤0.2	MIS	N

*Note:* N, variant is not in gnomAD v3.

Abbreviations: DNMs, de novo mutations; LOF, loss-of-function variant; MIS, missense variant; SV, structural variant; Y, yes.

^a^This proband also has an intragenic PUF60 deletion (see Table 2).

^b^This variant may be mosaic in the proband with ref:alt ratio 30:10. Genomic coordinates are based on GRCh38.

## Data Availability

The variant data used to support the findings of this study are included within the article and the supporting information.

## Supporting Information

Supporting Information 1 Table S1: CdLS categories (if available) for patients included in the analysis.

Supporting Information 2 Table S2: Candidate monoallelic (heterozygous or hemizygous) variants that survived filtering.

Supporting Information 3 Table S3: *NIPBL* noncoding variants identified in individuals affected with CdLS.

Supporting Information 4 Table S4: *NIPBL* 5⁣′ UTR noncoding variants reported in gnomAD.

Supporting Information 5 Figure S1: IGV plots of *NIPBL* coding region variants of probands and parents (where available). Figure S2: IGV plots of *NIPBL* intronic and splice site variants of probands and parents (where available). Figure S3: IGV plots of *ANKRD11* coding region variants of probands and parents (where available).
