# Supplementary material for: Whole Genome Sequencing of “Mutation-Negative” Individuals With Cornelia de Lange Syndrome
Source: Hum Mutat. 2025 Jan 30;2025:4711663. doi: 10.1155/humu/4711663 (PMC12267970; doi:10.1155/humu/4711663)
Supplement: Supporting Information 1 — Table S1: CdLS categories (if available) for patients included in the analysis. [file 4711663.f1.pdf]

Supplementary Table 1 - CdLS categories

Proband ID	Family ID	CdLS Classification	Height_SD	Weight_SD	OFC_SD
3027	3027	Mild	-2.27	0.01	-0.47
3028	3028	Mild	-1.15	-0.94	-1.21
3028	3028	Mild	-0.35		-1.49
3036	3036	Possible			
3037	3037	Atypical			
3040	3040	Possible			
3041	3041	Typical			
3046	3046	Possible			
3053	3053	Typical	-3.01	-3.03	-2.26
3057	3057	Possible	-1.98	0.35	0.27
3060	3060	Possible			
3177	3177	Typical			
3188	3188	Possible			
3236	3236	Typical		-2.32	
3379	3379	Possible	-2.29	-2.31	
3461	3461	Possible	0.14	1.64	-3.68
3471	3471	Possible			-0.26
3616	3616	Atypical	-0.84	-0.77	-3.93
3617	3617	Mild	-2.06	-0.97	-1.95
3778	3778	Uncertain			
3961	3961	Atypical	-2.49	-2.55	-5.05
4021	4021	Atypical			
4075	4075	Typical		-4.93	-5.29
4079	4079	Possible	-4.04		-4.76
4187	4187	Possible			
4197	4197	Typical	-4.36	-5.18	-7.24
4252	4252	Possible		-3.06	-0.18
4252	4252	Uncertain	-3.43	-3.03	
4281	4281	Possible			
4294	4294	Possible			-0.8
4306	4306	Typical			
4348	4348	Typical			
4353	4353	Mild	-1.11	-0.03	-2.64
4383	4383	Possible	-3.02	-2.73	-0.61
4427	4427	Mild	-3.4	-2.02	-2.31
4441	4441	Possible			
4445	4445	Typical			-4.21
4462	4462	Typical	-4.45	-9.91	-5.42
4485	4485	Typical	-1.18	-2.58	-2.66
4497	4497	Typical	-3.7	-2.76	-3.11
4536	4536	Uncertain			
4665	4665	Mild	-3.63	-1.67	-2
4691	4691	Typical			-4.87

4709	4709	Atypical			
4722	4722	Atypical	-1.5	-2.74	-3.78
4753	4753	Atypical	0.6	-0.53	0.22
5263	5263	Possible	-4.83	-8.07	-6.93
5320	5320	Typical		-5.29	
5651	5651	Atypical		-3.63	-4.87
5661	5661	Typical			
