# Supplementary material for: Whole Genome Sequencing of “Mutation-Negative” Individuals With Cornelia de Lange Syndrome
Source: Hum Mutat. 2025 Jan 30;2025:4711663. doi: 10.1155/humu/4711663 (PMC12267970; doi:10.1155/humu/4711663)
Supplement: Supporting Information 2 — Table S2: Candidate monoallelic (heterozygous or hemizygous) variants that survived filtering. [file 4711663.f2.pdf]

Supplementary Table 2 - Candidate monoallelic (heterozygous or hemizygous) variants that survived filtering

Family	Gene	DDG2P	De novo	Variant(s) of note	Amino acid variant	Variant type	In gnomAD	Additional comments (DRF)	Pathogenicity prediction
3379	ANKRD11	Y	?	chr16(GRCh38):g.89280521del NM_013275.5(ANKRD11):c.6021delC p.(Phe2008Serfs*79)	2008	LOF	N	Likely clinical diagnosis	Pathogenic
3471	ANKRD11	Y	?	chr16(GRCh38):g.89284635_89284639del NM_013275.5(ANKRD11):c.1903_1907del p.(Lys635Glnfs*26)	635	LOF	N	Likely clinical diagnosis	Pathogenic
4252	ANKRD11	Y	Y	chr16(GRCh38):g.89281638_89281639insGC NM_013275.5(ANKRD11):c.4903_4904insGC p.(Leu1635Argfs*52)	1635	LOF	N	Likely clinical diagnosis	Pathogenic
4294	ANKRD11	Y	Y	chr16(GRCh38):g.89282611G>A NM_013275.5(ANKRD11):c.3931C>T p.(Arg1311*)	1311	LOF	N	Likely clinical diagnosis	Pathogenic
4348	ANKRD11	Y	?	chr16(GRCh38):g.89284130_89284134del NM_013275.5(ANKRD11):c.2408_2412del p.(Lys803Argfs*5)	803	LOF	N	Likely clinical diagnosis	Pathogenic
4753	ANKRD11	Y	Y	chr16(GRCh38):g.89284364_89284367del NM_013275.5(ANKRD11):c.2175_2178del p.(Asn725Lysfs*23)	725	LOF	Y	Looks mosaic in gnomAD, ClinVar have it as LP, one allele in individual >70 yrs ? Benign clonal expansion	Pathogenic
3471	ARID1B	Y	?	chr6(GRCh38):156778617:156778617:T:G:ENST00000636930.2:c.937T>G; ENSP00000490491.2:p.Phe313Val; SIFT: Deleterious low confidence (0); PolyPhen: Probably damaging (0.997); CADD23.2; REVEL0.051; SpliceAI≤ 0.2			N	REVEL and CADD not strong	Not LP/P
4497	ARID1B	Y	?	NM_001346813.1(ARID1B):c.65_66del p.(Glu22Glyfs*209) low level mosaic. Not confirmed by an alternative assay.	22	LOF	N	De novo	Not LP/P
4847	ARID3A	N	Y	chr19(GRCh38):964425-964425: ENST00000263620.8:c.944G>A; ENSP00000263620.2:p.Arg315Gln; SIFT: Deleterious (0.02); PolyPhen: Probably damaging (0.993); CADD32; REVEL0.72; SpliceAI≤ 0.2	315	MIS	N	De novo, high pLi, no other reports	Possible novel locus
4414	ASXL1	Y	?	chr20(GRCh38):g.32434844C>T ENST00000375687.10:c.2132C>T; ENSP00000364839.4:p.Thr711Ile; SIFT: Tolerated low confidence (0.12); PolyPhen:Benign (0.029); CADD8.717; REVEL0.073; SpliceAI≤ 0.2	711	MIS	Y	Inheritance unknown	Not LP/P
3027	CACNA1A	Y	?	chr19(GRCh38):13275893:13275893:C:T; ENSP00000353362.5:p.Asp1316Asn; Sift: Deleterious (0); PolyPhen: Possibly damaging (0.655); CADD27.6; REVEL0.94; SpliceAI≤ 0.2	1316	MIS	N	near cluster of LP/P missense, no clinical fit	Not LP/P
4665	CASK	Y	?	chrX(GRCh38):41626688:41626688:C:T:HEM:ENST00000378163.7:c.931G>A; ENSP00000367405.1:p.Ala311Thr; SIFT: Deleterious (0.04); PolyPhen: Benign (0.065); CADD23.7; REVEL0.313; SpliceAI≤ 0.2	311	MIS	N	Inheritance unknown	Not LP/P
3062	DDX3X	Y	?	chrX(GRCh38):41341583:41341583:T:G:HEM:ENST00000644876.2:c.251T>G; ENSP00000494040.1:p.Phe84Cys; SIFT: Tolerated (0.15); PolyPhen: Benign (0); CADD22.9; REVEL0.16; SpliceAI≤ 0.2			Y	No support	Not LP/P
4383	EBF3	Y	Y	chr10(GRCh38):129877825-129877825: ENST00000440978.2:c.579G>T; ENSP00000387543.2:p.Lys193Asn; SIFT: Deleterious (0); PolyPhen: Probably damaging (1); CADD28; REVEL0.437; SpliceAI≤ 0.2	193	MIS	N	De novo, recurrent mutation	Pathogenic
4441	EFTUD2	Y	?	chr17(GRCh38):44859931:44859931:T:G:HET:ENST00000426333.7:c.1834A>C; ENSP00000392094.1:p.Lys612Gln; SIFT: Deleterious (0.01); PolyPhen: Probably damaging (0.973); CADD28.7; REVEL0.78; SpliceAI≤ 0.2	612	MIS	N	Inheritance unknown, microcephaly noted	Uncertain
4462	EHMT1	Y	Y	NM_024757.4(EHMT1):c.3695_3715dup p.(Leu1238_Gly1239insVEAGEQL)	1238	MIS	N	De novo	Pathogenic
4187	EHMT1	Y	?	chr9(GRCh38):g.137752355dup NM_024757.4(EHMT1):c.1195dupC p.(Gln399Profs*14)	399	LOF	N	Inheritance unknown	Pathogenic

3778	EMC1	Y	?	chr1(GRCh38):19243668:19243668:G:A:ENST00000477853.6:c.326C>T; ENSP00000420608.1:p.Ser109Phe; SIFT: Deleterious (0); PolyPhen: Probably damaging (0.999); CADD31; REVEL0.316; SpliceAI≤ 0.2	109	MIS	N	Inheritance unknown, monoallelic varants reported but with cerebellar atrophy as major component, REVEL not strong but CADD high	Uncertain
4306	EP300	Y	?	chr22(GRCh38):41126006:41126006:A:G:HET:ENST00000263253.9:c.872A>G; ENSP00000263253.7:p.Lys291Arg; SIFT: Tolerated (0.42); PolyPhen: Probably damaging (0.948); CADD22.7; REVEL0.318; SpliceAI≤ 0.2	291	MIS	Y	Inheritance unknown, missense predictions not supportive	Not LP/P
3961	EP300	Y	?	chr22(GRCh38):41166649:41166649:A:G:ENST00000263253.9:c.3857A>G; ENSP00000263253.7:p.Asn1286Ser; SIFT: Deleterious (0); PolyPhen: Probably damaging (0.985); CADD33; REVEL0.608; SpliceAI; ΔS donor gain 0.92; ΔS donor loss 0.4	1286	MIS	N	Inheritance unknown, likely splice variant	Likely Pathogenic
3037	EP300	Y	?	chr22(GRCh38):g.41177730_41177731del NM_001429.3(EP300):c.6019_6020del p.(Gln2007Valfs*65)	2007	LOF	N	Likely clinical diagnosis	Pathogenic
3188	EP300	Y	?	chr22(GRCh38):g.41162780G>C NM_001429.3(EP300):c.3728+1G>C p.?		ESS	N	Likely clinical diagnosis	Pathogenic
4507	IFIH1	Y	?	chr2(GRCh38):162268237:162268237:A:G:HET; NM_022168.4(IFIH1):c.2657T>C p.(Met886Thr); SIFT=deleterious(0.0):PolyPhen=possibly damaging(0.554):	886	MIS	Y	Inheritance unknown, implausible MAF	Not LP/P
3043	KCNQ2	Y	?	chr20(GRCh38):63414092:63414092:C:T:HET:ENST00000359125.7:c.1627G>A; ENSP00000352035.2:p.Val543Met; SIFT: Deleterious (0.02); PolyPhen: Probably damaging (0.936); CADD25; REVEL0.774; SpliceAI≤ 0.2			Y	No seizures seems unlikely	Not LP/P
3236	KMT2A	Y	?	chr11(GRCh38):g.118484286dup NM_001197104.1(KMT2A):c.4190dup p.(Val1398Serfs*9)	1398	LOF	N	Likely clinical diagnosis	Pathogenic
4485	MCM7	N	Y	chr7(GRCh38):100098712-100098712 chr7(GRCh38)g.100098712G>A ENST00000303887.10 c.586C >T p.Gln196Ter mosaic Looks real: ref G 30, alt A 10	196	LOF	N	De novo, no disease association with this gene, pLi 0	Possible novel locus
3057	MED13L	Y	?	chr12(GRCh38):g.115966244C>A NM_015335.4(MED13L):c.6226-1G>T p.?		ESS	N	DECIPHER 291493	Pathogenic
4353	MIS18BP1	Y	Y	(NM_018353.4):c.1833_1840+3delinsAACC, p.(Lys612Thrfs*14)			N	De novo, no disease association with this gene	Possible novel locus
3052	MYT1	Y	?	Chr20(GRCh38):g.64212121C>G ENST00000328439.6:c.1500C>G; ENSP00000327465.1:p.Asn500Lys; SIFT: Deleterious (0); PolyPhen: Probably damaging (0.992); CADD26.1; REVEL0.245; SpliceAI≤ 0.2			N	REVEL and CADD not strong, no clinical fit	Not LP/P
4021	NEDD4L	Y	?	chr18(GRCh38):58323243:58323243:G:A:ENST00000400345.8:c.422G>A; ENSP00000383199.2:p.Arg141Gln; SIFT: Deleterious (0.01); PolyPhen: Possibly damaging (0.729); CADD29.3; REVEL0.252; SpliceAI≤ 0.2			Y	Inheritance unknown, associated with PVNH	Not LP/P
4079	NIPBL	Y	Y	chr5(GRCh38):36876791C>T:ENST00000282516.13:c.-467C>T; CADD20.1; SpliceAI≤ 0.2		NC	N	De novo	Possible
4197	NIPBL	Y	Y	chr5(GRCh38):37052633-37055017 chr5(GRCh38):37055031-37055942 ~3.6 Kb SV inv or inv_dup in NIPBL ex42-43 region		DEL	N	De novo	Likely Pathogenic
4281	NIPBL	Y	Y	chr5(GRCh38):g.36955508C>T NM_133433.3(NIPBL):c.101C>T p.(Ala34Val)	34	MIS	N	De novo	Pathogenic
4427	NIPBL	Y	Y	chr5(GRCh38):37012200-37012200: ENST00000282516.13:c.4560+1975G>C; CADD1.325; SpliceAI≤ 0.2		NC	N	De novo	Possible
4445	NIPBL	Y	Y	chr5(GRCh38):g.37014728C>T NM_133433.3(NIPBL):c.4606C>T p.(Arg1536*) mosaic: ref 25, alt 3	1536	LOF	N	De novo	Pathogenic
4497	NIPBL	Y	Y	last 2 or 3 exons (exons 45-47, del of approx. 37061000-37067000) and downstream chr5(GRCh38):37072385>G>C		DEL	N	De novo	Pathogenic
4691	NIPBL	Y	Y	chr5(GRCh38):g.37022050G>C NM_133433.3(NIPBL):c.5329-1G>C p.? Chr5(GRCh38):g.37022050G>C		ESS	N	De novo	Pathogenic
4709	NIPBL	Y	Y	chr5(GRCh38):36876936-36876937del: ENST00000282516.13:c.-321del 5_prime_UTR_variant SpliceAI≤ 0.2		NC	N	De novo	Possible
4722	NIPBL	Y	Y	chr5(GRCh38):g.37011272C>G NM_133433.4(NIPBL):c.4560+1047C>G AND chr5(GRCh38):g.37011947C>A NM_133433.4(NIPBL):c.4560+1722C>A AND chr5(GRCh38):g.37012195G>T NM_133433.4(NIPBL):c.4560+1970G>T Intron 21		NC	N	De novo	Possible

5263	NIPBL	Y	Y	chr5(GRCh38):g.37060974del NM_133433.3(NIPBL):c.7816del p.(Ile2606Serfs*5)	2606	LOF	N	De novo	Pathogenic
5320	NIPBL	Y	Y	chr5(GRCh38):g.37026228G>A NM_133433.3(NIPBL):c.5710-1G>A p.? Chr5(GRCh38):g.37026228G>A		ESS	N	De novo	Pathogenic
5651	NIPBL	Y	Y	chr5(GRCh38):g.37010177del NM_133433.3(NIPBL):c.4512del p.(Leu1504Phefs*85) chr5(GRCh38):g.37010177del	1504	LOF	N	De novo	Pathogenic
3616	NIPBL	Y	?	chr5(GRCh38):g.36985329_36985330del NM_133433.3(NIPBL):c.2149_2150del p.(Lys717Glufs*2) mosaic: ref 32, alt 3	717	LOF	N	Inheritance unknown	Pathogenic
4536	NIPBL	Y	?	chr5(GRCh38):g.37017018G>A NM_133433.3(NIPBL):c.4777-1G>A p.?		ESS	N	Inheritance unknown	Pathogenic
3177	NIPBL	Y	?	chr5(GRCh38):g.37036491A>C ENST00000282516.13:c.5971+4A>C; CADD21.2; SpliceAI≤ 0.2		ESS	N	SpliceAI not supportive, inheritance unknown, typical CdLS	Possible
4021	NLGN3	Y	?	chrX(GRCh38):71167650:71167650:G:A:HEM: NM_181303.2(NLGN3):c.1553G>A p.(Trp518*)	518	LOF	N	Inheritance unknown, likely to be DECIPHER 279406	Likely Pathogenic
3046	NR2F1	Y	?	chr5(GRCh38):93585427:93585427:G:A:ENST00000327111.8:c.404G>A; ENSP00000325819.3:p.Arg135His; SIFT: Deleterious (0); PolyPhen: Probably damaging (1); CADD32; REVEL0.962; SpliceAI≤ 0.2	135	MIS	N	Likely clinical diagnosis	Likely Pathogenic
3461	NUP210	N	Y	chr3(GRCh38):13360354-13360354: ENST00000254508.7:c.2070C>G; ENSP00000254508.5:p.Ile690Met; SIFT: Deleterious (0.01); PolyPhen: Possibly damaging (0.533); CADD17.58; REVEL0.047; SpliceAI≤ 0.2			N	De novo, no support	Not LP/P
4248	PHIP	Y	?	chr6(GRCh38):78946244:78946243:-:T:HET; chr6(GRCh38):g.78946244dup NM_017934.7(PHIP):c.4387dup p.(Arg1463Lysfs*35)	1463	LOF	N	Inheritance unknown, see PMID: 31337854	Likely Pathogenic
3060	PIK3C3	N	Y	chr18(GRCh38):41957625-41957625C>T; ENST00000262039.9:c.124C>T; ENSP00000262039.3:p.Pro42Ser; SIFT: Deleterious (0); PolyPhen: Probably damaging (0.971); CADD25.4; REVEL0.66; SpliceAI≤ 0.2	42	MIS	N	De novo, no strong support PMID: 24038936, PMID: 27607605	Possible novel locus
3043	POLD1	Y	?	chr19(GRCh38):50402637:50402637:A:G:ENST00000440232.7:c.866A>G; ENSP00000406046.1:p.Asp289Gly; SIFT: Deleterious (0.01); PolyPhen: Probably damaging (0.925); CADD26.4; REVEL0.413; SpliceAI≤ 0.2			Y	Unlikely	Not LP/P
4353	PUF60	Y	Y	chr8(GRCh38):143820938-143823597 deletes exons 3 and 4 PUF60		DEL	N	De novo	Likely Pathogenic
4507	RAI1	Y	?	chr17(GRCh38):17797774:17797774:C:T:HET; NM_030665.4(RAI1):c.4826C>T p.(Thr1609Ile); SIFT=deleterious(0.0):PolyPhen=probably damaging(0.928):	1609	MIS	Y	Inheritance unknown, implausible MAF	Not LP/P
4044	SCN11A	Y	?	chr3(GRCh38):38847388:38847388:A:T:ENST00000302328.9:c.4682T>A; ENSP00000307599.3:p.Leu1561Gln; SIFT: Deleterious (0.03); PolyPhen: Probably damaging (0.995); CADD27.3; REVEL0.879; SpliceAI≤ 0.2			N	Inheritance unknown, this gene associated with episodic pain	Not LP/P
4482	SET	Y	Y	Heterozygous deletion chr9(GRCh38):128678964-128699851		DEL	N		Likely Pathogenic
3036	SETD5	Y	?	chr3(GRCh38):g.9441638del NM_001080517.1(SETD5):c.856del p.(Leu286*)	286	LOF	N	Likely clinical diagnosis	Pathogenic
5661	SMC1A	Y	Y	chrX(GRCh38):53405788-53405788: ENST00000322213.9:c.1714C>T; ENSP00000323421.3:p.Pro572Ser; SIFT: Deleterious (0.01); PolyPhen: Probably damaging (0.996); CADD24.8; REVEL0.86; SpliceAI≤ 0.2;	572	MIS	N	De novo	Pathogenic
3617	STAG1	Y	?	chr3(GRCh38):136472477:136472477:T:C:ENST00000383202.7:c.1141A>G; ENSP00000372689.2:p.Met381Val; SIFT: Deleterious (0); PolyPhen: Possibly damaging (0.601); CADD25.6; REVEL 0.721; SpliceAI≤ 0.2	381	MIS	N	Inheritance unknown, close to a cluster of pathogenic missense variants	Uncertain
3053	TBL1XR1	Y	?	chr3(GRCh38):177038113:177038113:G:T:HET:ENST00000457928.7:c.1107C>A; ENSP00000413251.3:p.Asp369Glu; SIFT: Deleterious (0.02); PolyPhen: Probably damaging (0.985); CADD23.7; REVEL0.379; SpliceAI≤ 0.2	369	MIS	N	DDD-WOS / Patient 260528 in cluster of LP/P missense see PMID: 26740553	Likely Pathogenic
4954	WDR18	N	Y	chr19(GRCh38):991291-991291: ENST00000585809.6:c.871G>A; ENSP00000476117.3:p.Glu291Lys; SIFT: Tolerated (0.12); PolyPhen: Possibly damaging (0.498); CADD25.6; REVEL0.268; SpliceAI≤ 0.2	291	MIS	N	De novo, high pLi, no other reports	Possible novel locus
