# Supplementary material for: Whole Genome Sequencing of “Mutation-Negative” Individuals With Cornelia de Lange Syndrome
Source: Hum Mutat. 2025 Jan 30;2025:4711663. doi: 10.1155/humu/4711663 (PMC12267970; doi:10.1155/humu/4711663)
Supplement: Supporting Information 3 — Table S3: NIPBL noncoding variants identified in individuals affected with CdLS. [file 4711663.f3.pdf]

Supplementary Table 3 - *NIPBL* non-coding variants identified in individuals affected with CdLS

CHROM	POS	REF	ALT	Feature	Variant	Consequence	Kozak	Details	ClinVar ID	ClinVar significance	REF DOI	In this study
5	36876791	C	T	ENST00000282516	c.-467C>T	uAUG_gained	Strong	156bp long uORF	1195876	Pathogenic/Likely_pathogenic	10.3390/genes13050740	Y
5	36876801	GA	AT	ENST00000282516	c.-457_-456delinsAT	uAUG_gained	Moderate	270bp long uORF	1300231	Likely_pathogenic	10.1002/humu.24384	
5	36876937	CC	A	ENST00000282516	c.-321_-320delinsA	uORF_frameshift	Moderate	uORF extended from 15 to 189bps	2151	Pathogenic	10.1002/humu.20380	
5	36877164	C	T	ENST00000282516	c.-94C>T	uAUG_gained	Weak	51bp long uORF	NA		10.1002/humu.24384; 10.1111/j.1399-0004.2007.00832.x	
5	36876936	GC	G	ENST00000282516	c.-315del	uORF_frameshift	Moderate	uORF extended from 15 to 189bps	NA		NA	Y
