# Supplementary material for: Whole Genome Sequencing of “Mutation-Negative” Individuals With Cornelia de Lange Syndrome
Source: Hum Mutat. 2025 Jan 30;2025:4711663. doi: 10.1155/humu/4711663 (PMC12267970; doi:10.1155/humu/4711663)
Supplement: Supporting Information 4 — Table S4: NIPBL 5⁣′ UTR noncoding variants reported in gnomAD. [file 4711663.f4.pdf]

Supplementary Table 4 - *NIPBL* 5' UTR non-coding variants reported in gnomAD

CHROM	POS	REF	ALT	AC	Feature	Variant	Consequence	Kozak	Details
5	36876946	T	A	427	ENST00000282516	c.-312T>A	uSTOP_lost	Moderate	
5	36876976	G	A	1	ENST00000282516	c.-282G>A	uAUG_gained	Weak	117bp long uORF
5	36876984	A	C	1	ENST00000282516	c.-274A>C	uAUG_lost	Weak	
5	36876984	A	G	1	ENST00000282516	c.-274A>G	uAUG_lost	Weak	
5	36876994	AGGAG	A	2	ENST00000282516	c.-262_-259del	uORF_frameshift	Weak	uORF extended from 90 to 108bps
5	36877040	G	A	1	ENST00000282516	c.-218G>A	uSTOP_gained	Weak	
5	36877059	T	A	1	ENST00000282516	c.-199T>A	uAUG_lost	Weak	
5	36877073	A	AG	1	ENST00000282516	c.-183dup	uORF_frameshift	Weak	uORF reduced from 66 to 39bps
5	36877097	CA	C	1	ENST00000282516	c.-160del	uORF_frameshift	Weak	uORF extended from 66 to 141bps
5	36877098	A	AC	7	ENST00000282516	c.-153dup	uORF_frameshift	Weak	uORF extended from 66 to 165bps
5	36877098	AC	A	7	ENST00000282516	c.-153del	uORF_frameshift	Weak	uORF extended from 66 to 141bps
5	36877118	T	TTATA	3	ENST00000282516	c.-139_-136dup	uORF_frameshift	Weak	uORF extended from 66 to 168bps
5	36877123	G	C	1	ENST00000282516	c.-135G>C	uSTOP_lost	Weak	
5	36877172	C	CAT	1	ENST00000282516	c.-86_-85insAT	uAUG_gained	Weak	48bp long uORF
5	36953628	A	G	2	ENST00000282516	c.-69A>G	uAUG_lost	Strong	
