# Supplementary material for: Whole Genome Sequencing of “Mutation-Negative” Individuals With Cornelia de Lange Syndrome
Source: Hum Mutat. 2025 Jan 30;2025:4711663. doi: 10.1155/humu/4711663 (PMC12267970; doi:10.1155/humu/4711663)

Figure S1. IGV plots of *NIPBL* coding regions variants of probands and parents (where available)

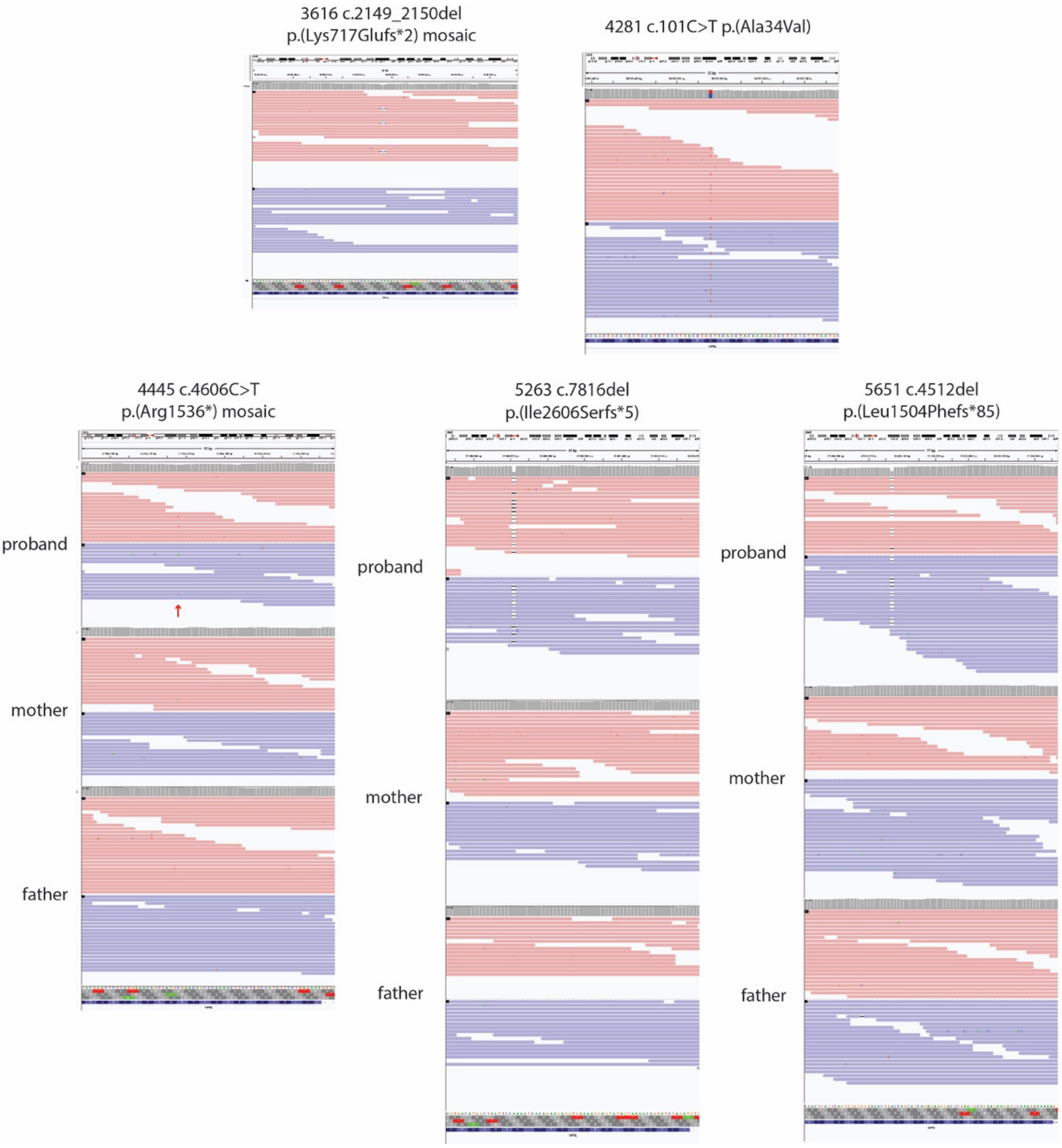

Figure S2. IGV plots of *NIPBL* essential splice site variants of probands and parents (where available)

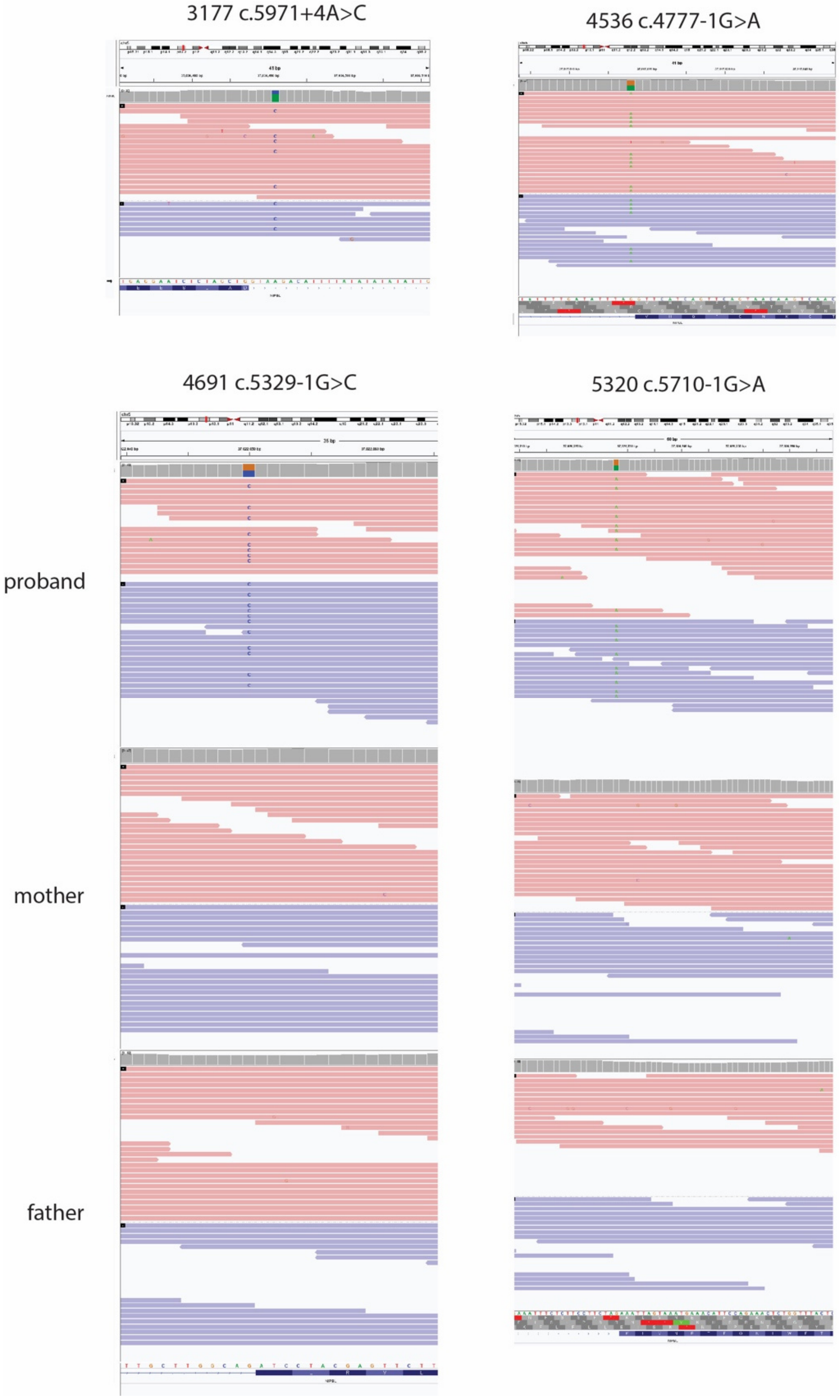

Figure S3. IGV plots of *ANKRD11* coding regions variants of probands and parents (where available)

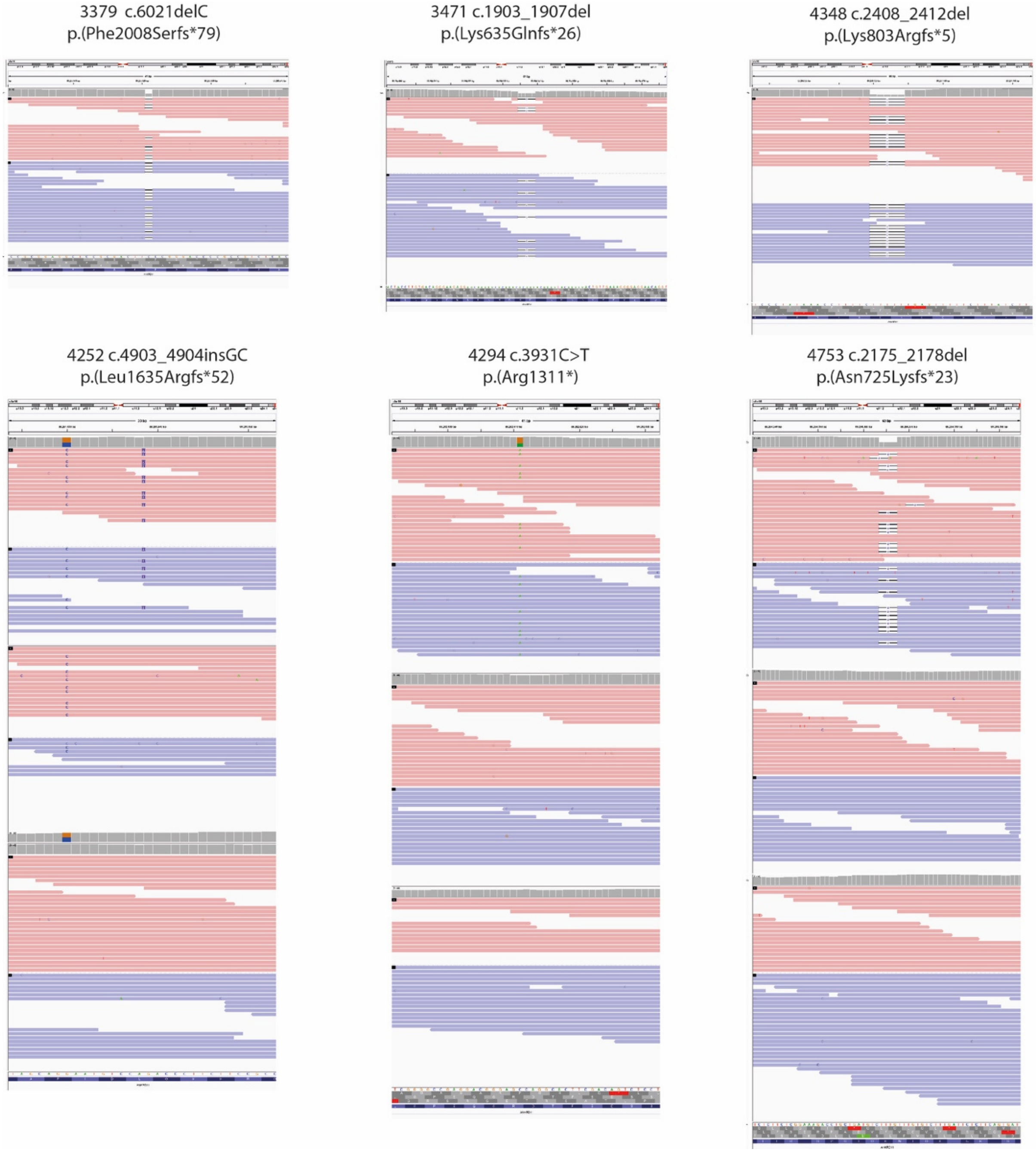

Supplement: Supporting Information 5 — Figure S1: IGV plots of NIPBL coding region variants of probands and parents (where available). Figure S2: IGV plots of NIPBL intronic and splice site variants of probands and parents (where available). Figure S3: IGV plots of ANKRD11 coding region variants of probands and parents (where available). [file 4711663.f5.pdf]
